# Discovery of Novel Inhibitor for WNT/β-Catenin Pathway by Tankyrase 1/2 Structure-Based Virtual Screening

**DOI:** 10.3390/molecules25071680

**Published:** 2020-04-06

**Authors:** Bo Li, Jinxia Liang, Feng Lu, Guandi Zeng, Jindao Zhang, Yinxing Ma, Peng Liu, Qin Wang, Qian Zhou, Liang Chen

**Affiliations:** 1 Institute of Life and Health Engineering, Jinan University, Guangzhou 510632, China; 2 School of Life sciences, Lanzhou University, Lanzhou 730000, China

**Keywords:** tankyrase 1/2 inhibitors, WNT/β-catenin signaling pathway, virtual screening, dock, antitumor

## Abstract

Aberrant activation of the WNT/β-catenin signaling pathway is implicated in various types of cancers. Inhibitors targeting the Wnt signaling pathway are intensively studied in the current cancer research field, the outcomes of which remain to be determined. In this study, we have attempted to discover novel potent WNT/β-catenin pathway inhibitors through tankyrase 1/2 structure-based virtual screening. After screening more than 13.4 million compounds through molecular docking, we experimentally verified one compound, LZZ-02, as the most potent inhibitor out of 11 structurally representative top hits. LiCl-induced HEK293 cells containing TOPFlash reporter showed that LZZ-02 inhibited the transcriptional activity of β-catenin with an IC_50_ of 10 ± 1.2 μM. Mechanistically, LZZ-02 degrades the expression of β-catenin by stabilizing axin 2, thereby diminishing downstream proteins levels, including c-Myc and cyclin D1. LZZ-02 also inhibits the growth of colonic carcinoma cell harboring constitutively active β-catenin. More importantly, LZZ-02 effectively shrinks tumor xenograft derived from colonic cell lines. Our study successfully identified a novel tankyrase 1/2 inhibitor and shed light on a novel strategy for developing inhibitors targeting the WNT/β-catenin signaling axis.

## 1. Introduction

The highly conserved WNT/β-catenin signaling pathway plays pivotal roles in the context of development, differentiation and cellular self-renewal [1,2,3]. Not surprisingly, aberrant activation of WNT/β-catenin signaling has been implicated in various types of cancers [4,5]. Therefore, the identification of a novel inhibitor targeting the WNT/β-catenin pathway for cancer treatment is imperatively needed in the current onco-clinic. However, the limited numbers of identified inhibitors targeting the Wnt/β-catenin signaling pathway restrain the scope by which the proteins of the Wnt/β-catenin signaling pathway could be targeted.

Being important activators of the WNT/β-catenin pathway in colon cancer, tankyrase is a hot topic for drug development and promising clinical data have been reported for several tankyrase-targeting reagents as anti-cancer drugs [6,7]. The abnormal activation of tankyrase stabilized cellular β-catenin through the degradation of axin proteins are the crucial components of the β-catenin destruction complex [8,9]. Tankyrase belong to the family of proteins known as poly (ADP)-ribose polymerases (PARPs), consisting of two members: tankyrase 1 (TNKS-1) and tankyrase 2 (TNKS-2), sharing 85% of amino acid sequence identity [10,11] and a conserved catalytic ADP-riosyltransferases (ARTD) domain [12]. In addition to their roles in the WNT/β-catenin pathway, TNKS-1/2 are also involved in telomere length maintenance and mitosis. The overexpression of TNKS-1/2 has been reported to induce telomere elongation in human cancer cells [13,14]. Inhibiting the activity of tankyrases is expected to target two of the most common pathways important for tumor cell survival: (1) telomere shortening [15]; (2) β-catenin degradation by stabilizing axin [16,17,18] and the inhibition of the transcription of important cell proliferating genes of the WNT/β-catenin pathway targets, such as *c-Myc* [19] and *Cyclin D1* [20]. These studies suggest tankyrase is a highly attractive target to develop small molecular inhibitors for cancer treatment [21,22].

In 2009, Huang and colleagues characterized a small molecule inhibitor of TNKS-1/2, XAV939, as the first potent inhibitor of TNKS-1/2 with IC_50_ values in the nanomolar range [23]. Since then multiple potent scaffolds resembling XAV939 have been reported to target the same catalytic domain, including flavones [24,25], arylnaphthyridinones [25], 2-Arylquinazolin-4-ones [26], and MSC2504877 [27]. Other structurally different inhibitors have also been reported to impair WNT signaling in vitro by targeting tankyrase, including IWR-1/2 [28], JW74/55 [17,29], WIKI4 [30], G007-LK [31], with some validated for anti-tumor efficacy in xenograft and/or genetically engineered mouse models of cancer [32]. However, in most cases, high doses of tankyrase inhibitors required to suppress tumor growth often result in intestinal toxicity, weight loss and even death in rodents. Safer and more effective tankyrase inhibitors are urgently needed in clinic.

Screening approaches relying on biological activity; chemical genetic screen [23], high-throughput transcriptional screening technology [33] or TOPFlash reporter assay [34] are highly powerful ways to identify the novel tankyrases inhibitors. However, the costly and time-consuming procedures limit their applications in the ultra-high throughput screening of large chemical libraries. Recently, the drug discovery process has been accelerated with the aid of computer-assisted drug design (CADD) [35]. Molecular docking programs rate chemicals based on the free energy of the complex of chemical-target protein, and thus enables the structure-based virtual screening of large compound databases for inhibitors against a protein of interest [36,37]. The crystal structure of the catalytic domain of TNKS-1/2 has been resolved, making it possible for structure-based design and development of tankyrase inhibitor scaffolds [38,39]. TNKS-1/2 play an important role by utilizing NAD^+^ as a substrate to generate ADP-ribose polymers. The donor NAD^+^ binding groove includes two sub-sites: nicotinamide (NI) and the adenosine (ADE). Depending on the targeting site, the tankyrase inhibitors can be categorized as: NI subsite targeting (such as XAV939) [23] and ADE subsite targeting (such as IWR-1) [40,41]. Several novel tankyrase inhibitors have been defined by structure- or ligand-based virtual screening. However, most studies are limited to WNT/β-catenin pathway downregulation, with biological effects, including those on cell growth, remaining largely to be determined [42,43,44,45,46].

In this study, we conducted the TNKS-1/2 structure-based virtual screening. We validated the best hit, LZZ-02, as a novel TNKS-1/2 inhibitor. LiCl-induced HEK293 cells containing TOPFlash reporter showed LZZ-02 (IC_50_ = 10 ± 1.2 μM) to be an effective WNT inhibitor. Mechanistically, LZZ-02 stabilized axin 2 and thus degraded β-catenin proteins. Moreover, LZZ-02 exerted potent antitumor activity against DLD1-derived colorectal tumor xenograft tumor. Our study highlights promising anticancer application of LZZ-02 and suitability as a lead candidate for further developing WNT/β-catenin inhibitors.

## 2. Results and Discussion

### 2.1. Preparation for Structure-Based Virtual Screening

The human tankyrase protein family consists of TNKS-1 and TNKS-2, featuring a catalytic ARTD domain at the C-terminus of 89% of overall sequence identity. The structure has been resolved for the TNKS inhibitor development [38,47,48]. The crystal structures of TNKS-2 in a complex with XAV939 revealed that the tankyrase inhibitor interacts with the NAD^+^ binding groove of the catalytic domain [49].

**Figure 1:**
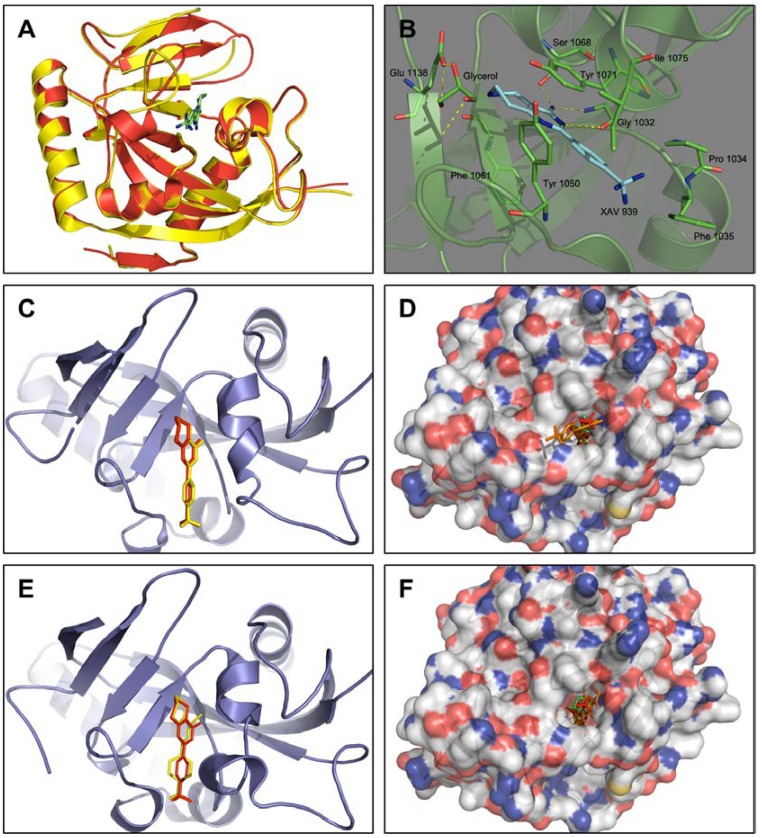
Identify binding site and evaluate docking program. (**A**): comparison of crystal structure of TNKS-1 (yellow, 2RF5) and TNKS-2 (red, 3KR8), XAV939 shows its original location in co-crystal with TNKS-2 (green). (**B**): Several amino acids were involved in the interaction with XAV939 in the active site pocket of TNKS-2, hydrogen bonds are represented by dashed lines. (**C**): XAV939 were re-docked back into the active site of TNKS-2 (red) by *DOCK6.5* and the pose of XAV939 were at the original position in co-crystal (yellow). (**D**): XAV939 (red), ABT-888 (green), LDW643 (orange) were docked into the same activated pocket in TNKS-2 by *DOCK6.5*. (**E**): XAV939 were re-docked by *Autodock4.2* (red) and the pose of XAV939 were original position in co-crystal (yellow). (**F**): XAV939 (red), ABT-888 (green), LDW643 (orange) were docked in TNKS-2 by *Autodock4.2*. All graphical pictures were generated using the *PyMol* program (http://www.pymol.org/).

We retrieved crystal structures of TNKS-1 (PDB: 2RF5) and TNKS-2 (PDB: 3KR8). The co-crystallized inhibitor XAV939 occupies the whole nicotinamide binding region of TNKS-2, which was referenced to construct the grids for docking screening (Figure 1). TNKS-1 displayed a similar substrate-binding and overall 3D structure to TNKS-2. Our simulation revealed that it has the same targeting region as TNKS-2 (Figure 1A). Prior to screening the ZINC database, evaluation of the accuracy of the docking programs, *DOCK6.5* and *Autodock4.2*, was necessary. The re-docking test is an experimental method to evaluate the reproducibility of a complex structure by docking calculation and it has always been used to evaluate the performance of a docking program [50,51,52]. The docking programs were considered to qualify for virtual screening if the root mean square deviation (RMSD) for the ligand between the docked conformation and the crystallographic conformation was < 2 Å [53,54]. Herein, the TNKS-2 protein bound original ligand XAV939 was re-docked into the binding pocket by *DOCK6.5* or *Autodock4.2*, the RMSD of the ligand between obtained the docked pose. We found that the RMSD was only 0.2 Å. Moreover, similar patterns were confirmed with three known TNKS-2 inhibitors when docked into the same area by *DOCK6.5* and *Autodock4.2*, respectively (Figure 1C). Taken together with the above data, it showed that *DOCK6.5* and *Autodock4.2* were suitable for our virtual screening. 

The flexible docking program could improve the accuracy of screening due to its capacity to more closely mimic the protein in nonrigid residues, although at the price of longer computing time. The co-crystal structures of TNKS-2 with XAV939 revealed that some residues are important for inhibitors to occupy the catalyzing site: the Gly1032 and Ser1068 form hydrogen bonds with XAV939; Tyr1071 also shown a π-π stacking interaction with it [39]. Therefore, we selected residues around XAV939 including Tyr1071, Ser1068, Gly1032, Phe1061, Tyr1050, Tyr1071, Pro1034, Phe1035 and Ile1075 as flexibility residues in our flexible docking screening (Figure 1D).

Superimposing known inhibitors has been reported to serve as a screening criterion in the virtual screening process to improve accuracy and effectively avoid false positive compounds. We selected three of the experimentally validated TNKS-1/2 inhibitors reported by Huang et al. [23] (XAV939, ABT-888 and LDW643) as the reference compounds in our screening (Table S1). IWR-1-endo and IWR-1-exo were excluded due to the fact that they bound in a site other than the catalytic domain [55].

### 2.2. Discovery of Candidate Compounds by Virtual Screening

**Figure 2:**
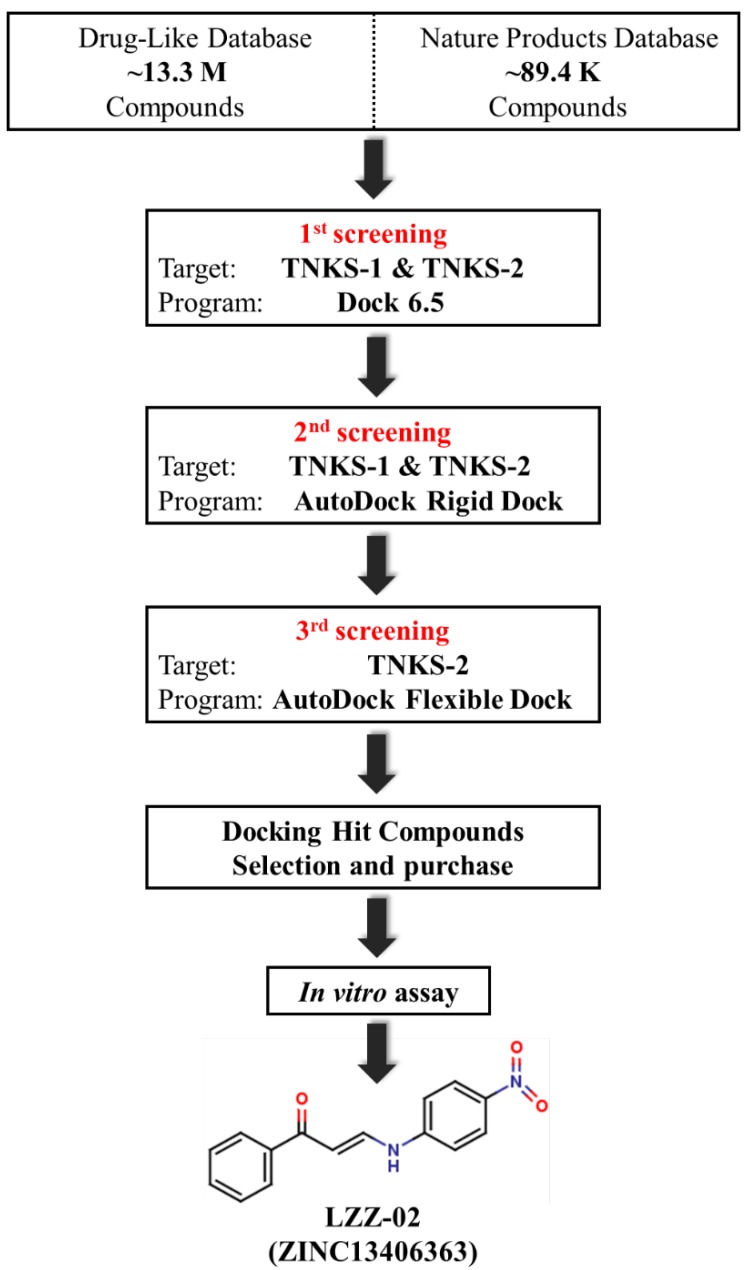
General workflow for the discovery of the TNKS-1/2 inhibitor.

Following the above process, we screened the drug-like and natural product databases in ZINC (http://zinc.docking.org/) of more than 13.4 million compounds through three steps in silico (Figure 2). Prior to each virtual screening, the three reference compounds were used to test whether the dock and score system gave results that were consistent with the biological assay. We only collected the compounds with scores better than LDW643. 

Given the volume of compounds in the database, the rapid docking program and simple scoring evaluation for efficiency were the chief goal in the primary screening. Since *DOCK6.5* is fast for matching compounds to a specific site on target protein and also utilizes a relatively simple scoring function to evaluating van der waals (VDW) and electrostatic interactions, it was employed for the first round of screening. First, the compounds were docked in tankyrase 2; chemicals with scores below −40 kcal/mol were chosen for docking into tankyrase 1; subsequently, the compounds which scored below −25 kcal/mol were chosen for the second round of docking (Table S2). 

We performed the second round of more accurate screening with *Autodock4.2*, a program scoring the docking of chemical base on the Lamarckian Genetic Algorithm. The compounds were likewise first docked into tankyrase 2 and collected the chemicals which scored below −10 kcal/mol for docking into tankyrase 1; subsequently, compounds with a score less than −7 kcal/mol were subjected to further screening. 

At the final step, a flexible ligand-protein docking program was used for precisely filtering compounds and those which scored below −8.5 kcal/mol were selected. After removing duplicate compounds and excluding incorrect and similar structures, 326 compounds were obtained as the hit compounds (Table S3). We visually inspected the hit-list and purchased 11 structurally representative compounds for further validation (Table 1).

### 2.3. Biological Evaluation of the Purchased Compounds

**Figure 3:**
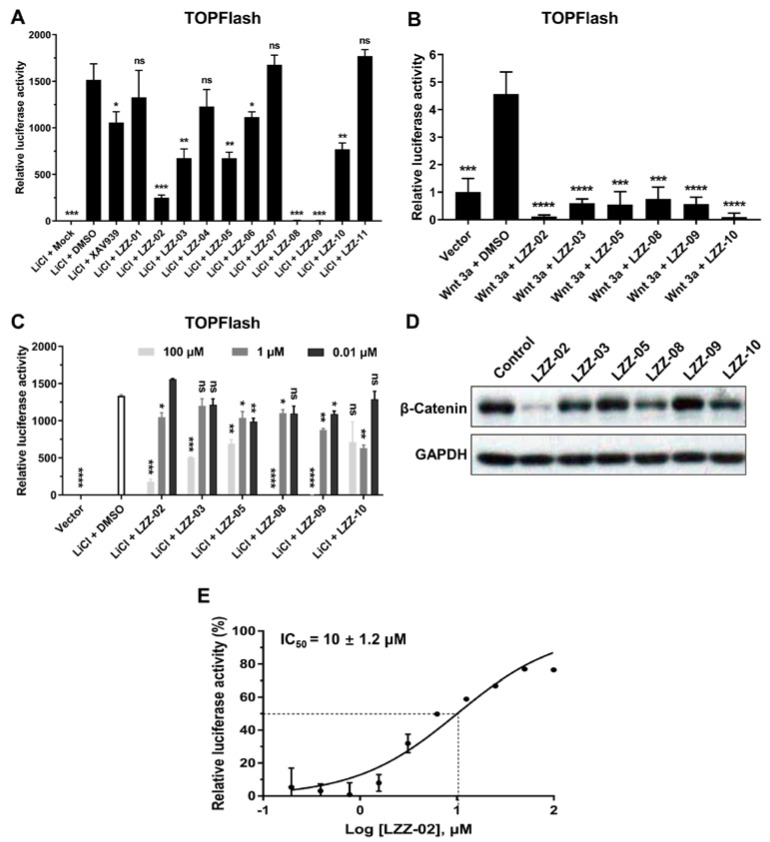
Evaluation of hits as tankyrase inhibitors. (**A**): Biological screening of candidates. HEK293 cells (1 × 10^5^) were transfected with the indicated reporter plasmid (0.1 μg) and then further treated with the indicated candidates’ compounds (100 μM) and LiCl for 24 h before luciferase assays were performed. (**B**): The compounds inhibit WNT 3a-induced TOPFlash activity. HEK293 cells (1 × 10^5^) were transfected with the indicated reporter plasmid (0.1 μg). Cells were then left untreated or treated with the indicated candidates’ compounds (100 μM) and Wnt 3a for 24 h before luciferase assays were performed. (**C**): The experiments were similarly performed as in (A), LZZ-02 inhibited LiCl-upregulated TOPFlash activity in dose-dependent. (**D**): Effects of the candidates’ compounds on β-catenin expression in DLD1 cells. DLD1 (2 × 10^6^) cells were left untreated or treated with indicated compounds (100 μM). Cell lysates were analyzed by immunoblots with the indicated antibodies. (**E**): Transfected cells treated with LZZ-02 at indicated concentration. Data are presented as mean (n = 3) ± SD, ns: non-significant, * *p* < 0.05, ** *p* < 0.01, *** *p* < 0.001, **** *p* < 0.0001.

We used a 9X TCF luciferase reporter gene assay (TOPFlash) to functionally validate the ability of chemicals to inhibit the β-catenin transcription activity in HEK293 cells [23]. We found that LiCl and Wnt3a strongly enhanced the transcription activity of β-catenin, indicating the authenticity of our system to validate the ability of a chemical for its ability to target the WNT/β-catenin pathway. Interestingly, six compounds (LZZ-02, LZZ-03, LZZ-05, LZZ-08, LZZ-09 and LZZ-10) significantly inhibited both Wnt3a- and LiCl-upregulated TOPFlash activity in this screening (Figure 3A–C). All of the six compounds were well docked into the same sites as shown in Figure S1.

DLD-1 cells harbor an APC truncation, rendering β-catenin stabilization and constitutive expression of downstream target genes [56]. To further validate the inhibitory activity of these compounds against the WNT pathway, we checked the protein level of β-catenin in DLD-1 cells treated with the above chemicals respectively. Strikingly, a significant decrease of β-catenin protein level was seen in cells treated with LZZ-02, LZZ-08 and LZZ-10. Of note, LZZ-02 showed the strongest inhibitory effect on β-catenin protein level in DLD1 cells (Figure 3D). Moreover, assaying the activity of the TOPFlash reporter in transfected cells revealed an IC_50_ value showed around 10 ± 1.2 μM (Figure 3E). Therefore, we focused our further experimental efforts on LZZ-02. 

### 2.4. LZZ-02 Suppresses WNT/β-Catenin Signaling by Increasing Axin 2 Protein Level

**Figure 4:**
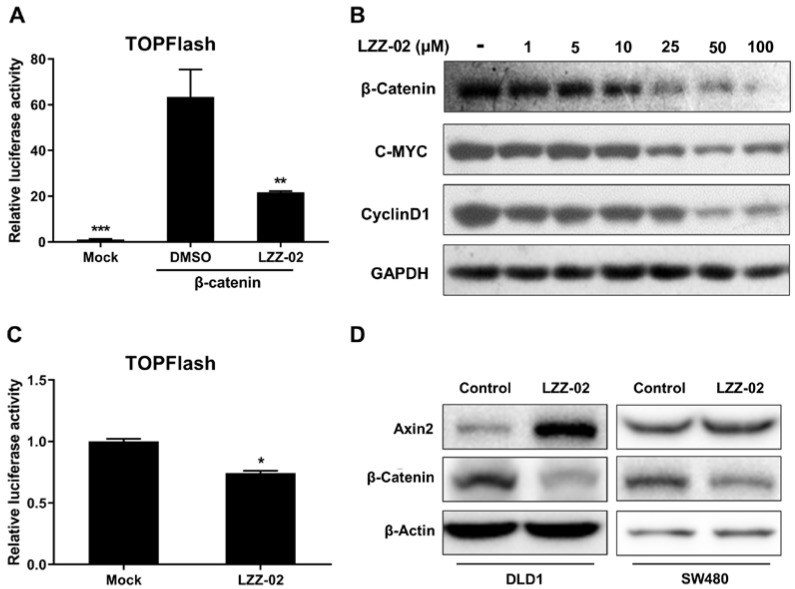
Tankyrase inhibitor LZZ-02 suppresses WNT/β-catenin signaling by increasing axin 2 protein level. (**A**): LZZ-02 inhibits the β-catenin induced TOPFlash activity in HEK293 cells. HEK293 cells (1 × 10^5^) were transfected with the reporter plasmid (0.1 μg) and β-catenin plasmid (0.1 μg) for 12 h. Cells were then left untreated or treated with LZZ-02 for 24 h before luciferase assays were performed. (**B**): LZZ-02 restrains β-catenin and WNT downstream protein expression in a dose-dependent manner. DLD1 (2 × 10^6^) were left untreated or treated with LZZ-02 with an indicated concentration for 24 h, and cell lysates were analyzed by immunoblots with the indicated antibodies. (**C**): LZZ-02 inhibit TOPFlash activity in DLD1 cells. DLD1 cells (1 × 10^5^) were transfected with the TOPFlash reporter plasmid (0.2 μg), and then further treated with LZZ-02 for 24 h before luciferase assays were performed. (**D**): LZZ-02 increases axin 2 protein and reduces β-catenin level in human colorectal cancer cells. DLD1 cells (2 × 10^6^) were left untreated or treated with indicated compounds (30 μM). Cell lysates were analyzed by immunoblots with the indicated antibodies. Data are presented as mean (n = 3) ± SD, * *p* < 0.05, ** *p* < 0.01, *** *p* < 0.001.

Tankyrase inhibitors regulate WNT signaling by increasing the level of axin 2, the scaffold protein and main component of the β-catenin destruction complex [23]. We went further to examine whether LZZ-02 was able to suppress the β-catenin induced WNT signaling pathway. The luciferase assay indicated that LZZ-02 restrains β-catenin mediated TOPFlash activity in HEK293 cells (Figure 4A). Aberrant activation of the WNT/β-catenin pathway promotes target proto-oncogenes *c-Myc* and *Cyclin D1* transcription, thereby increasing their protein level [19,20]. We found that LZZ-02 decreased the protein level of β-catenin, c-Myc and CyclinD1 in a dose-dependent manner (Figure 4B). In addition, TOPFlash assays suggested that LZZ-02 inhibits constitutive WNT activity in colon cancer cells DLD1 (Figure 4C). LZZ-02 is also able to increase axin 2 protein level and strongly decreased total β-catenin level in DLD1 cells. Meanwhile, the expression level of β-catenin was also reduced by LZZ-02 in SW480 cells (Figure 4D). Collectively, these data suggested LZZ-02 suppressed WNT/β-catenin signaling by increasing axin 2 protein level. 

### 2.5. LZZ-02 Inhibits the Growth of Colon Cancer Cells In Vitro

**Figure 5:**
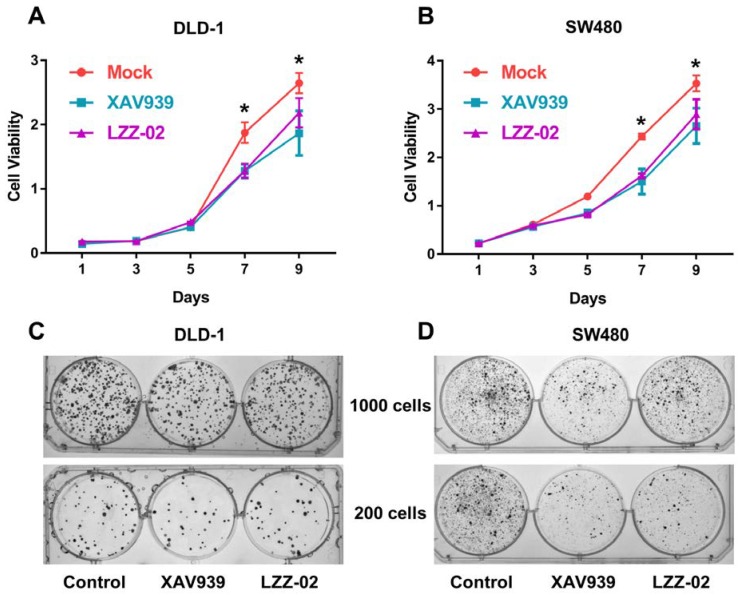
LZZ-02 and XAV939 inhibits cell proliferation of human colorectal cancer cells. (**A**,**B**): human colorectal cancer cell viability was significantly reduced upon LZZ-02 or XAV939 compound treatment. DLD-1 (**A**) and SW480 (**B**) cell line (500 cells) were treated without or with WNT inhibitors (30 μM), and ell viability was detected by a CCK-8 assay kit for the indicated time points. (**C**,**D**): LZZ-02 or XAV939 inhibits colony forming of human colorectal cancer cells. DLD-1 (**C**) and SW480 (**D**) cell line (200–1000 cells) were treated without or with WNT inhibitors (30 μM) for two weeks, and the cells were stained with 0.5% crystal violet. Representative images of the colony-forming assay of DLD1 and SW480 cells. Data are presented as mean (n = 3) ± SD. * *p* < 0.05.

The WNT/β-catenin signaling cascade plays an important role in cell proliferation [57]. XAV939 has been shown to reduce the proliferation of human colorectal cancer cells [29,58]. Herein, we asked whether LZZ-02 could inhibit the growth of colorectal cancer cell-harboring, aberrantly-active tankyrases. Interestingly, CCK8 assay revealed that LZZ-02 significantly inhibited the proliferation of DLD1 and SW480. Moreover, LZZ-02 has a similar inhibition efficiency to XAV939 (Figure 5A,B). Meanwhile, a dose-dependent decrease of cell numbers was also noted in DLD-1 and SW480 in the presence of LZZ-02 (Figure S2). More importantly, LZZ-02 also decreased the numbers of the colony forming of DLD1 and SW480 (Figure 5C,D). Considering that APC mutation and the aberrant activation of the WNT/β-catenin are common in colorectal cancer, LZZ-02 deserved further characterization.

### 2.6. LZZ-02 Inhibits the Growth of Subcutaneous DLD1 Xenografts

**Figure 6:**
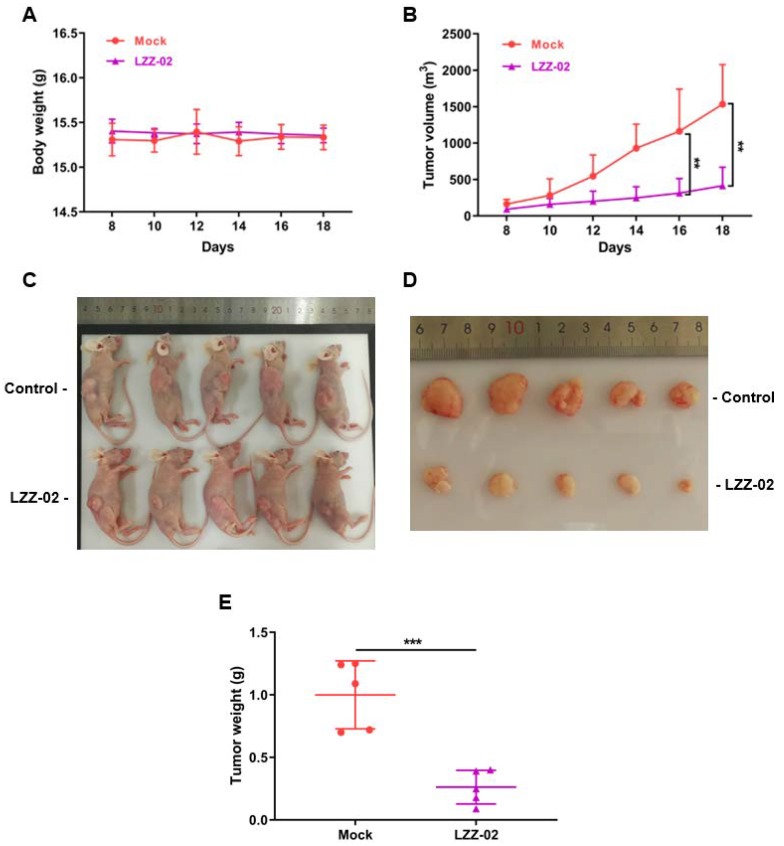
LZZ-02 inhibits the growth of DLD1 derived colorectal tumor xenograft model. DLD1 cells (2.5 × 10^6^) were implanted subcutaneously into the flank of 6-week-old female BALB/c nude mice. When the tumors reached a volume of about 80 mm^3^, animals were randomized into two groups (n = 5 each group): vehicle group and LZZ-02 group. Mice were orally gavaged every day for 18 days. (**A**): Body weight was recorded every three days and LZZ-02 does not affect the mice’s body weight. (**B**): Tumor growth was recorded every two days by measuring its diameter with Vernier caliper and the growth of LZZ-02 treated tumors were strongly suppressed compared control tumors in control group. (**C**): LZZ-02 does not affect the body weight of mice. (**D**,**E**): LZZ-02 treated tumor growth was drastically inhibited relative to the control group. Data are presented as mean ± SD. ** *p* < 0.01, *** *p* < 0.001.

To validate the antitumor activity of LZZ-02 in vivo, we inoculated DLD1 cells subcutaneously in flanks of nude mice. When tumors reached a volume of around 80 mm^3^, mice were randomized into two groups for treatment through oral gavage with 30 mg/kg of LZZ-02 or vehicle for 18 days. Interestingly, treatment with LZZ-02 caused significant tumor shrinkage (Figure 6B). At day 18 post treatment, the average weight of tumor in LZZ-02- treated group was around 0.26 g, in stark contrast to an average of around 1 g in the control group (Figure 6D,E). We also found that LZZ-02 is well tolerated by mice as judged by their constant body-weight (Figure 6A,C), indicative of favorable in vivo toxicity profile of LZZ-02.

### 2.7. Docking Study by Molecular Modeling of Interactions Between LZZ-02/XAV939 and TNKS-1/2

**Figure 7:**
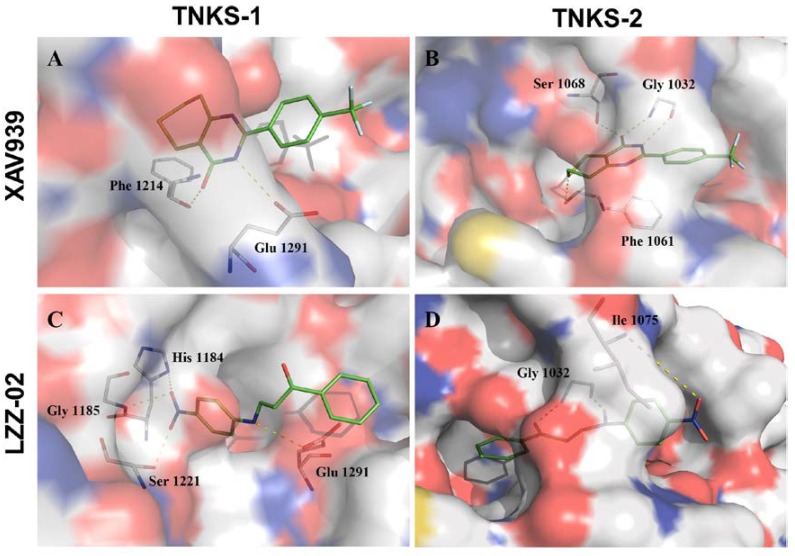
Binding mode of compounds XAV939, LZZ-02 in the active site of TNKS-1 (PDB: 2RF5) (**A**,**C**) and TNKS-2 (PDB: 3KR8) (**B**,**D**). Hydrogen bond was represented with yellow dashed line. All graphical picture was generated using *PyMol* program (http://www.pymol.org/).

We went further to check the detail of binding mode of LZZ-02 with TNKS-1/2. We found that LZZ-02 nested in the NAD^+^ pockets of TNKS-1/2 in a similar way to that of XAV939 (Figure 7A–D). Simulation analysis revealed that XAV939 anchored and formed four H-bonds in the binding site of TNKS-2: The pyrimidine nitrogen and hydroxyl and the sulfur atom in the thiopyrano ring were within hydrogen bonding distance of the Gly1032, Ser1068 and Phe1061 backbone, respectively (Figure 7B), consistent with a previous study [39]. Interestingly, LZZ-02 forms three H-bonds with TNKS-2, except the Gly1032 side chain stack with carbonyl and amino. Of note, nitro of LZZ-02 formed interactions with Ile1075 backbone near the opening of the binding crevice (Figure 7D). Similarly, LZZ-02 also formed a hydrogen bond with a Glu1291 side chain on the verge of the binding site of TNKS-1; its nitro group interacted with His1184, Gly1185 and Ser1221 backbones deep in the of pocket (Figure 7C). In contrast, XAV939 only formed interactions with Phe1214 and Glu1291 inside of TNKS-1 (Figure 7A). Compared with XAV939, LZZ-02 formed more additional interaction with amino acid near the edge of the binding crevice in TNKS-1/2, which could stabilize their binding position and thereby enhance the inhibitory effects, as suggested in an earlier report to optimize tankyrase inhibitors [39].

## 3. Materials and Methods

### 3.1. Virtual Screening

#### 3.1.1. Receptor Preparation

The 3D protein structure for docking was based on the X-ray crystal structure of the PARP domain of tankyrase. Free Tankyrase 1 and tankyrase 2 bound to its complex inhibitor, XAV939, 2-[4-(trifluoromethyl) phenyl]-7,8-dihydro-5H-thiopyrano [4,3-d] pyrimidin-4-ol], were obtained from the Protein Data Bank (PDB entry 2RF5 [38], 3KR8 [39], respectively). Prior to docking with *DOCK6.5*, the protein was fixed by deleting waters molecules, adding hydrogens and adding the Gasteiger charge. Before docking with *Autodock4.2*, the *MGL Tools* was used to treat proteins: adding the Gasteiger charge, adding the polar hydrogens, removing the water and writing in the pdbqt format.

#### 3.1.2. Ligand Preparation

The XAV939, ABT-888 and LDW6433D structures of these compounds were constructed using the Sketch Molecule module in the *SYBYL* software (Version 6.9, Tripos Associates, St. Louis, MO, USA). Energy minimization was performed by the Powell gradient algorithm with the Tripos force field [58] and the Gasteiger-Huckel charge [59]. More than 13.4 million structurally diverse compounds were downloaded from all-purchasable subsets of the publicly accessible ZINC database, which contains two databases: the Drug-Like Database of 13.3 million molecules and the Nature Products Database of 89.4 thousand molecules [60,61]. These compounds are selected because they are commercially available and they were filtered by applying Lipinski’s rule of five. All compounds were downloaded in MOL2 format, then added hydrogens and charge for virtual screening with *DOCK 6.5* directly. For docking with *Autodock*, compounds were papered with *prepare_ligand4.py* script in *Autodock tools*.

#### 3.1.3. Virtual Screening

The molecular docking program *DOCK 6.5* [62] was utilized to perform the first round virtual screening owing to its fast calculating speed, followed by the rigid dock program and the flexible docking using *Autodock 4.2* software, respectively. The job was performed on a Dawning A620-F cluster of 16 processors-each 2.6G AMD Opteron at the Gansu Computational Center.

### 3.2. Biological Evaluation

#### 3.2.1. Reagents, Constructs and Antibodies

Lipofectamine 2000, and dual-specific luciferase assay kit (Promega), ECL Western Blotting Substrate (Millipore), Cell Counting Kit-8 (Dojindo), chemical compounds were purchased from Topscience (Shanghai, China), other compounds come from the J&K company (Beijing, China). SW480, DLD-1, and HEK293 were obtained from ATCC (VA, USA). Mouse antibodies against β-actin (Sigma), GAPDH (Abcam). Rabbit antibodies against c-Myc (Abcam), axin 2 (CST), β-catenin (CST), Cyclin D1 (CST). Super TOPFlash (ST-Luc) (9 X TCF binding sites, β-catenin were constructed by standard molecular biology techniques. 

#### 3.2.2. Cell Culture

HEK293, DLD-1 and SW480 cells were cultured in DMEM (Dulbecco’s modified Eagle’s Medium) (Gibco, Shanghai, China) or RPMI 1640 Medium (Gibco, Shanghai, China), supplemented with 10% FBS (fetal bovine serum) (Gibco, Shanghai, China). 100 units/mL penicillin, and 100 mg/mL streptomycin and incubated at 37 °C in a 5% CO_2_ incubator (ThermoFisher, Shanghai, China).

#### 3.2.3. Transfection and Reporter Assay

HEK293 cells were transfected by the standard calcium phosphate precipitation method, and DLD1 cells were transfected with lipofectamine 2000 according to manufacturer’s instruction. An empty amount of vector plasmid was served as control in each transfection. To normalize for transfection efficiency, 0.1 μg Renilla luciferase reporter plasmid was added to each transfection. Luciferase assays were performed using a dual-specific luciferase assay kit.

#### 3.2.4. Cell Proliferation Assay

Cells were seeded in 96-well plates at 1 × 10^3^ per well. Cell proliferation was evaluated using the Cell Counting Kit-8 according to the manufacturer’s instructions. Briefly, 10 µL of the CCK-8 solution were added to culture medium and incubated for 2 h, and the absorbance at a 450 nm wave length was determined.

#### 3.2.5. Colony Formation Assay

Cells were seeded in 6-well plates at 200 or 1000 per well and maintained in a medium containing 0.5% FBS. Sixteen hours later, LZZ-02 were added at the indicated concentrations. The medium was replenished every two days until colony formation was observed. The cells were then washed twice with PBS, fixed with cold methanol, and stained with 0.5% crystal violet.

#### 3.2.6. Immunoblot Analysis

The cells were washed twice with ice-cold PBS and then lysed with ice-cold RIPA lysis buffer. Lysates were kept on ice for 30 min and then centrifuged at 13,000 rpm for 5 min at 4 °C. The supernatant was subjected to 10% SDS/PAGE and followed by immunoblot analysis with the indicated antibodies.

#### 3.2.7. In vivo Xenograft Model

All mice were housed in a pathogen-free environment in Jinan University. All experimental protocols were approved by the Institutional Committee for Animal Care and Use at Jinan University. All animal work was performed in strict accordance with the approved protocol. DLD1 cells (2.5 × 10^6^) suspended in a 100 μL mixture of equal volumes of PBS and Matrigel were implanted subcutaneously into the flank of 6-week-old female BALB/c nude mice. When the tumors had reached a volume of about 80 mm^3^, the 12 mice were then randomly divided into two groups. Animals received the compound by oral gavage, whereas the control group received vehicle solution. LZZ-02 were orally gavaged at 30 mg/kg once daily for 18 consecutive days. Tumor volumes and the body weight of animals were measured every three days. Tumor volume (tumor volume (cm^3^) = D × d^2^/2, where D is the longest and d is the shortest diameter, respectively) were monitored once every three days after 18 days up to the end of the experiment.

#### 3.2.8. Statistical Analysis

Statistics were performed using GraphPad Prism 7.04, and the student t-test was used to compare differences between the two experimental groups. The data are presented as means ± SD and *p* < 0.05 was considered statistically significant.

## 4. Conclusions

In conclusion, tankyrase 1/2 structure-based virtual screening was performed successfully to identify inhibitors of the WNT/β-catenin pathway from the ZINC Database. In total, 11 compounds were selected to test their in vitro inhibitory activities against the WNT/β-catenin pathway. Among them, LZZ-02 showed the most significant inhibitory potency against LiCl-, Wnt3a- or β-catenin-induced TOPFlash activity in HEK293 cells. Meanwhile, LZZ-02 showed remarkable inhibition of TOPFlash activity in DLD-1 cells, which express constitutively activated β-catenin. Furthermore, LZZ-02 stabilized axin 2 protein level and restrain β-catenin, c-Myc and Cyclin D1 expression in DLD1 and SW480 cells. LZZ-02 also inhibits the colonic cancer cells’ viability and colony formation. In the whole process of treatment, the mice were active, LZZ-02 showed antitumor function in vivo and the bodyweight of mice remains constant after treated. LZZ-02 is therefore worthy of further characterization in clinic. Equally important, LZZ-02 could serve as a lead candidate for developing highly potent tankyrase inhibitors, but the precise mechanism requires more investigation.

## Figures and Tables

**Table 1:** The scoring and structures information of the selected 11 compounds.

Compounds	Structure	TNKS-2 Docking Score (kcal/mol)
**LZZ-01** **(ZINC36709370)**	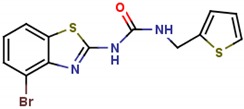	**−9.54**
**LZZ-02** **(ZINC13406363)**	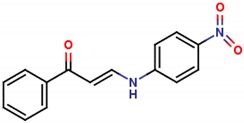	**−8.86**
**LZZ-03** **(ZINC00622715)**	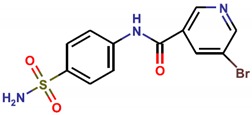	**−9.88**
**LZZ-04** **(ZINC04348886)**	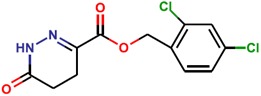	**−9.37**
**LZZ-05** **(ZINC18157250)**	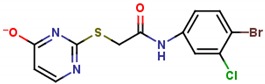	**−10.42**
**LZZ-06** **(ZINC08665842)**	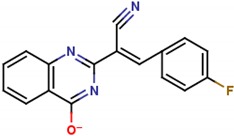	**−11.19**
**LZZ-07** **(ZINC00258875)**	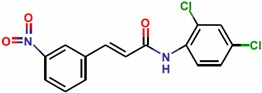	**−9.41**
**LZZ-08** **(ZINC04491425)**	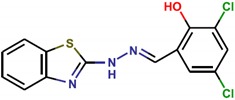	**−9.95**
**LZZ-09** **(ZINC01233403)**	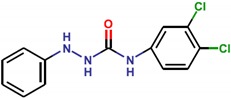	**−9.48**
**LZZ-10** **(ZINC00226990)**	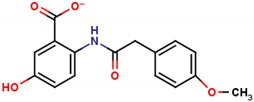	**−8.6**
**LZZ-11** **(ZINC00678564)**	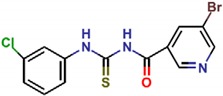	**−9.7**

## Supplementary Materials

Table S1. Known TNKS-1/2 inhibitors for virtual screening method validations. Table S2. The scoring results of training set compounds in each round screening. Table S3. The list of hit compounds by virtual screening (kcal/mol). Figure S1. Binding conformation of the selected 6 compounds. XAV939 as the positive control show similar conformation. Figure S2. LZZ-02 reduces proliferation in dose-dependent of human colorectal cancer cell. The DLD-1 (A) and SW480 (B) cell line were treated different concentration of LZZ-02.
