# Supplementary material for: Discovery of Novel Inhibitor for WNT/β-Catenin Pathway by Tankyrase 1/2 Structure-Based Virtual Screening
Source: Molecules. 2020 Apr 6;25(7):1680. doi: 10.3390/molecules25071680 (PMC7180783; doi:10.3390/molecules25071680)
Supplement: Supplementary file 1 [file molecules-25-01680-s001.pdf]

Table S1. Known TNKS-1/2 inhibitors for virtual screening method validations.

Compounds		Structur	IC_{50} (μM)	
			TNKS-1	TNKS-2
Positive compounds	XAV939	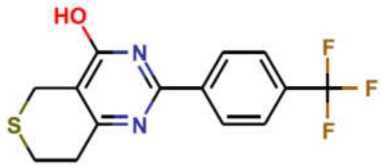	0.011	0.004
	ABT-888	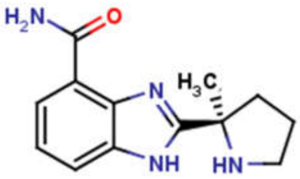	14.97	6.519
Negative compounds	LDW643	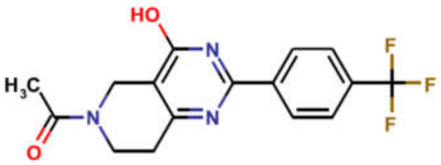	>18.75	>18.75

Table S2. The scoring results of training set compounds in each round screening.

	The first screening		The second screening		The third screening
**Program**	**Dock 6.5 (kcal/mol)**		**Autodock 4.2 (kcal/mol)**		
			**Rigid docking**		**Flexible docking**
**Target**	**TNKS-1**	**TNKS-2**	**TNKS-1**	**TNKS-2**	**TNKS-2**
**XAV939**	-23.3976	-48.7548	-7.27	-10.33	-9.15
**ABT-888**	-13.1766	-14.9015	-6.17	-9.61	-9.10
**LDW643**	4.0496	72.1593	-5.83	-8.17	-8.25
**Selection criteria**	Score <-25	Score <-40	Score < -7	Score < -10	Score < -8.5

Table S3. The list of hit compounds by virtual screening (kcal/mol).

No.	ZINC ID	Score	No.	ZINC ID	Score	No.	ZINC ID	Score
1	ZINC52797443	-11.77	2	ZINC49256676	-11.74	3	ZINC50076297	-11.62
4	ZINC48532432	-11.52	5	ZINC46707602	-11.41	6	ZINC49212160	-11.27
7	ZINC08665842	-11.19	8	ZINC49212158	-11.18	9	ZINC41494598	-11.16
10	ZINC41838563	-10.99	11	ZINC06523275	-10.97	12	ZINC00046136	-10.92
13	ZINC40464810	-10.89	14	ZINC48852264	-10.87	15	ZINC00558197	-10.82
16	ZINC30711763	-10.82	17	ZINC48460931	-10.8	18	ZINC04002868	-10.79
19	ZINC22276480	-10.79	20	ZINC02805543	-10.77	21	ZINC15841372	-10.77
22	ZINC04002868	-10.76	23	ZINC47178751	-10.75	24	ZINC08976550	-10.73
25	ZINC03410028	-10.71	26	ZINC14199385	-10.71	27	ZINC41503931	-10.71
28	ZINC17945479	-10.69	29	ZINC41494382	-10.68	30	ZINC21190150	-10.66
31	ZINC24684711	-10.66	32	ZINC40217444	-10.66	33	ZINC13540066	-10.65
34	ZINC00045867	-10.62	35	ZINC17945482	-10.59	36	ZINC46441514	-10.59
37	ZINC46706224	-10.59	38	ZINC41817520	-10.56	39	ZINC51476603	-10.55
40	ZINC16524412	-10.54	41	ZINC20478435	-10.54	42	ZINC00117759	-10.53
43	ZINC01633452	-10.53	44	ZINC19618576	-10.53	45	ZINC05897357	-10.52
46	ZINC41494476	-10.52	47	ZINC45145474	-10.52	48	ZINC48312042	-10.52
49	ZINC20457194	-10.51	50	ZINC19229951	-10.5	51	ZINC46440763	-10.5
52	ZINC08736925	-10.49	53	ZINC41540248	-10.49	54	ZINC46302999	-10.49
55	ZINC48789716	-10.49	56	ZINC05897522	-10.48	57	ZINC46440960	-10.48
58	ZINC19299726	-10.47	59	ZINC20501285	-10.46	60	ZINC41261592	-10.46
61	ZINC07682439	-10.45	62	ZINC29024748	-10.45	63	ZINC00403751	-10.44
64	ZINC13218630	-10.44	65	ZINC08013743	-10.43	66	ZINC36649351	-10.43
67	ZINC52634096	-10.43	68	ZINC18157250	-10.42	69	ZINC18154570	-10.42
70	ZINC48163626	-10.42	71	ZINC06883349	-10.4	72	ZINC48532834	-10.4
73	ZINC18916048	-10.39	74	ZINC23056157	-10.39	75	ZINC24764587	-10.39
76	ZINC06942491	-10.38	77	ZINC31662108	-10.38	78	ZINC36150225	-10.38
79	ZINC06565834	-10.37	80	ZINC47211461	-10.37	81	ZINC42625404	-10.36
82	ZINC00106579	-10.35	83	ZINC16545658	-10.35	84	ZINC04473189	-10.34
85	ZINC06128147	-10.34	86	ZINC05123806	-10.32	87	ZINC18045297	-10.32
88	ZINC44599448	-10.32	89	ZINC48511047	-10.32	90	ZINC01393082	-10.31
91	ZINC03513118	-10.31	92	ZINC03945150	-10.31	93	ZINC06173862	-10.31
94	ZINC06402594	-10.31	95	ZINC12234495	-10.31	96	ZINC35683920	-10.3
97	ZINC06232957	-10.29	98	ZINC29992439	-10.29	99	ZINC31738761	-10.29
100	ZINC32722157	-10.29	101	ZINC39558284	-10.28	102	ZINC44599505	-10.28
103	ZINC49513822	-10.28	104	ZINC06321701	-10.27	105	ZINC18916147	-10.27
106	ZINC32593744	-10.27	107	ZINC40641689	-10.27	108	ZINC46441871	-10.27
109	ZINC00482100	-10.26	110	ZINC12908231	-10.26	111	ZINC41546180	-10.26
112	ZINC24237232	-10.25	113	ZINC46440752	-10.25	114	ZINC52168652	-10.25
115	ZINC16046278	-10.24	116	ZINC48559522	-10.24	117	ZINC12969663	-10.22
118	ZINC12969663	-10.22	119	ZINC41819081	-10.22	120	ZINC20865231	-10.21
121	ZINC31739033	-10.21	122	ZINC01394404	-10.2	123	ZINC05900669	-10.2

124	ZINC21596072	-10.2	125	ZINC49662784	-10.2	126	ZINC48407429	-10.19
127	ZINC31257346	-10.18	128	ZINC42622932	-10.18	129	ZINC43982700	-10.18
130	ZINC46440554	-10.18	131	ZINC46440582	-10.18	132	ZINC47326869	-10.18
133	ZINC00450485	-10.17	134	ZINC14517990	-10.17	135	ZINC24234500	-10.17
136	ZINC24917945	-10.17	137	ZINC21015290	-10.16	138	ZINC48163624	-10.16
139	ZINC51508211	-10.16	140	ZINC01582618	-10.15	141	ZINC41545894	-10.15
142	ZINC41546118	-10.15	143	ZINC06484711	-10.15	144	ZINC47326892	-10.15
145	ZINC46023120	-10.14	146	ZINC48828333	-10.14	147	ZINC23188793	-10.13
148	ZINC03171666	-10.13	149	ZINC44789152	-10.13	150	ZINC42241225	-10.12
151	ZINC02808548	-10.12	152	ZINC13844228	-10.12	153	ZINC18193878	-10.11
154	ZINC00091693	-10.11	155	ZINC48012561	-10.11	156	ZINC41838581	-10.1
157	ZINC48823694	-10.09	158	ZINC32116581	-10.09	159	ZINC03671320	-10.08
160	ZINC47211483	-10.08	161	ZINC41851206	-10.07	162	ZINC09667597	-10.06
163	ZINC42649470	-10.06	164	ZINC05186981	-10.05	165	ZINC13324122	-10.05
166	ZINC37650746	-10.05	167	ZINC41494445	-10.04	168	ZINC08763411	-10.03
169	ZINC00467723	-10.02	170	ZINC41427391	-10.02	171	ZINC00061440	-10.01
172	ZINC03665097	-10.01	173	ZINC36110521	-10.01	174	ZINC20514010	-10.01
175	ZINC00311673	-10	176	ZINC45058232	-10	177	ZINC52772713	-9.99
178	ZINC30692595	-9.99	179	ZINC08747967	-9.99	180	ZINC12617881	-9.98
181	ZINC08380754	-9.97	182	ZINC44294845	-9.97	183	ZINC46023127	-9.97
184	ZINC01505326	-9.96	185	ZINC49470859	-9.96	186	ZINC17056571	-9.95
187	ZINC04491425	-9.95	188	ZINC40494199	-9.95	189	ZINC46180017	-9.94
190	ZINC09970544	-9.94	191	ZINC17994427	-9.94	192	ZINC48606614	-9.94
193	ZINC00260145	-9.93	194	ZINC30711344	-9.92	195	ZINC09190234	-9.91
196	ZINC33175045	-9.91	197	ZINC24365657	-9.9	198	ZINC47178589	-9.9
199	ZINC20501381	-9.89	200	ZINC44449712	-9.89	201	ZINC00622715	-9.88
202	ZINC48599780	-9.88	203	ZINC41790459	-9.87	204	ZINC51452714	-9.87
205	ZINC47365453	-9.86	206	ZINC12772325	-9.85	207	ZINC36796036	-9.85
208	ZINC00095022	-9.84	209	ZINC00364199	-9.84	210	ZINC08312770	-9.84
211	ZINC41496874	-9.84	212	ZINC17071336	-9.83	213	ZINC17303821	-9.82
214	ZINC44059479	-9.82	215	ZINC05961651	-9.81	216	ZINC41540170	-9.81
217	ZINC13327841	-9.8	218	ZINC45761384	-9.8	219	ZINC08874089	-9.79
220	ZINC18203869	-9.79	221	ZINC41606431	-9.79	222	ZINC17329856	-9.78
223	ZINC45786257	-9.78	224	ZINC41502332	-9.77	225	ZINC04912659	-9.76
226	ZINC25393269	-9.76	227	ZINC48939139	-9.76	228	ZINC05743391	-9.75
229	ZINC48441556	-9.75	230	ZINC16545596	-9.74	231	ZINC42630735	-9.74
232	ZINC03844748	-9.74	233	ZINC11639373	-9.73	234	ZINC45173744	-9.73
235	ZINC48231534	-9.72	236	ZINC04758584	-9.72	237	ZINC12744358	-9.72
238	ZINC44253082	-9.72	239	ZINC52375188	-9.72	240	ZINC28988991	-9.71
241	ZINC19795262	-9.7	242	ZINC00678564	-9.7	243	ZINC36987796	-9.69
244	ZINC01232385	-9.68	245	ZINC05177897	-9.68	246	ZINC20535234	-9.68
249	ZINC26779691	-9.67	248	ZINC43462495	-9.67	249	ZINC41495689	-9.66
250	ZINC03098078	-9.66	251	ZINC05152108	-9.66	252	ZINC18174715	-9.66

253	ZINC03331873	-9.64	254	ZINC46189750	-9.64	255	ZINC03295645	-9.63
256	ZINC01269775	-9.62	257	ZINC13685343	-9.62	258	ZINC00671201	-9.61
259	ZINC06377410	-9.61	260	ZINC02853861	-9.6	261	ZINC48461951	-9.6
262	ZINC49356352	-9.6	263	ZINC20501230	-9.59	264	ZINC32116363	-9.59
265	ZINC07050319	-9.57	266	ZINC41311355	-9.57	267	ZINC32118579	-9.56
268	ZINC38933968	-9.56	269	ZINC40287189	-9.56	270	ZINC01060885	-9.55
271	ZINC00138206	-9.54	272	ZINC01665922	-9.54	273	ZINC04808337	-9.54
274	ZINC31255091	-9.54	275	ZINC36709370	-9.54	276	ZINC00256382	-9.53
277	ZINC06863333	-9.53	278	ZINC18192083	-9.53	279	ZINC03984977	-9.53
280	ZINC37489202	-9.53	281	ZINC07914243	-9.51	282	ZINC36633231	-9.51
283	ZINC48388984	-9.51	284	ZINC06208304	-9.5	285	ZINC28809070	-9.5
286	ZINC01164123	-9.49	287	ZINC00678569	-9.48	288	ZINC01233403	-9.48
289	ZINC03403999	-9.48	290	ZINC05090183	-9.48	291	ZINC03231105	-9.47
292	ZINC20535874	-9.47	293	ZINC06218408	-9.46	294	ZINC32119018	-9.46
295	ZINC00652514	-9.45	296	ZINC17178772	-9.45	297	ZINC44078711	-9.45
298	ZINC48433553	-9.45	299	ZINC49467276	-9.45	300	ZINC16500343	-9.44
301	ZINC03322487	-9.43	302	ZINC19618742	-9.43	303	ZINC49100035	-9.43
304	ZINC05569817	-9.42	305	ZINC06061644	-9.42	306	ZINC47835379	-9.42
307	ZINC00258875	-9.41	308	ZINC07953215	-9.41	309	ZINC07263344	-9.4
310	ZINC09742648	-9.39	311	ZINC41899422	-9.39	312	ZINC36803126	-9.39
313	ZINC00904337	-9.38	314	ZINC04348886	-9.37	315	ZINC36288192	-9.36
316	ZINC24997512	-9.35	317	ZINC00408783	-9.35	318	ZINC31922095	-9.35
319	ZINC00200926	-9.34	320	ZINC11689968	-9.23	321	ZINC00276614	-8.95
322	ZINC04280945	-8.92	323	ZINC04349934	-8.89	324	ZINC13406363	-8.86
325	ZINC00139294	-8.73	326	ZINC00226990	-8.6			

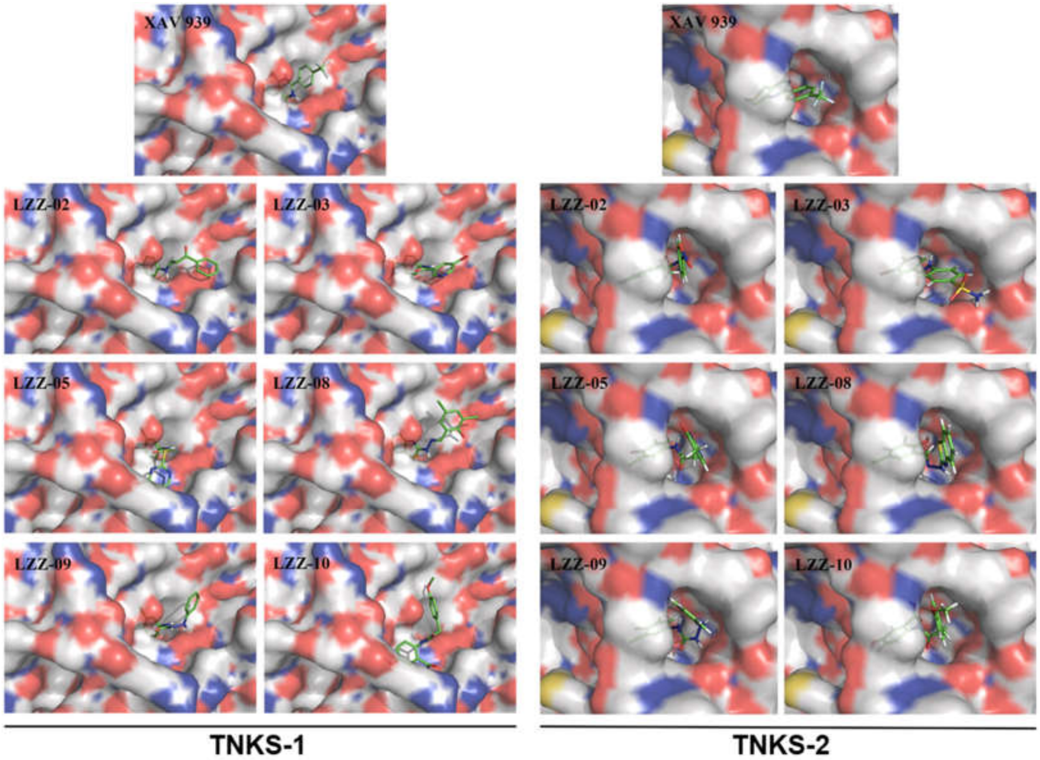

Figure S1. Binding conformation of the selected 6 compounds. XAV939 as the positive control show similar conformation.

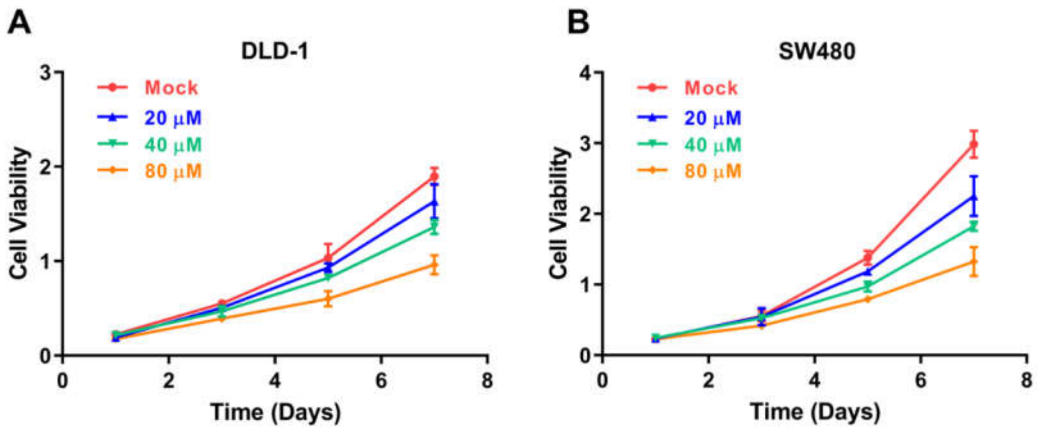

Figure S2. LZZ-02 reduces proliferation in dose-dependent of human colorectal cancer cell. The DLD-1 **(A)** and SW480 **(B)** cell line were treated different concentration of LZZ-02.
